# A phase I and pharmacokinetic study of indisulam in combination with carboplatin

**DOI:** 10.1038/sj.bjc.6603606

**Published:** 2007-02-06

**Authors:** C Dittrich, A S Zandvliet, M Gneist, A D R Huitema, A A J King, J Wanders

**Affiliations:** 1 Ludwig Boltzmann-Institute for Applied Cancer Research (LBI-ACR VIEnna) and ACR–ITR VIEnna, Third Medical Department – Centre for Oncology and Haematology, Kaiser Franz Josef-Spital, A-1100 Vienna, Austria; 2 Department of Pharmacy and Pharmacology, The Netherlands Cancer Institute/Slotervaart Hospital, NL-1066 EC Amsterdam, The Netherlands; 3 Eisai Ltd., 3 Shortlands, London, W6 8EE, UK

**Keywords:** indisulam, carboplatin, phase I, pharmacokinetics

## Abstract

Indisulam (E7070) is an anticancer agent that is currently being evaluated in phase II clinical studies. A significant reduction in glutathione synthetase and glutathione reductase transcripts by indisulam provided a molecular basis for its combination with platinum agents. Indisulam demonstrated high anti-tumour activity in various preclinical cancer models. The objectives of this study were (1) to determine the recommended dose of indisulam in combination with carboplatin in patients with solid tumours and (2) to evaluate the pharmacokinetics of the combination. Patients with solid tumours were treated with indisulam in combination with carboplatin. Indisulam (350, 500, or 600 mg m^−2^) was given as a 1-hour intravenous infusion on day 1 and carboplatin (5 or 6 mg min ml^−1^) as an intravenous infusion over 30 min on day 2 of a three-weekly cycle. Sixteen patients received study treatment and were eligible. Thrombocytopenia was the major dose limiting toxicity followed by neutropenia. Both drugs contributed to the myelosuppressive effect of the combination. Indisulam 500 mg m^−2^ in combination with carboplatin 6 mg min ml^−1^ was identified not to cause dose limiting toxicity, but a delay of re-treatment by 1 week was required regularly to allow recovery from myelosuppression. The recommended dose and schedule for an envisaged phase II study in patients with non-small cell lung cancer is indisulam 500 mg m^−2^ in combination with carboplatin 6 mg min ml^−1^ repeated four-weekly. Patients who do not experience severe thrombocytopenia at cycle 1 will be permitted to receive an escalated dose of indisulam of 600 mg m^−2^ from cycle 2 onwards.

Indisulam (E7070) is a chloro-indolyl sulphonamide anticancer agent that induces dose- and time-dependent G1 cell-cycle arrest in addition to delay in G1/S transition and S-phase progression (Fukuoka *et al*, 2001). Significant downregulation of particular sets and subsets of genes involved in cell-cycle control and energy metabolism was observed. Inhibition of carbonic anhydrase isoform IX (CAIX), which was initially supposed to be the main mechanism of action does not appear to be able to explain the whole aspects of indisulam anti-tumour activity (Owa *et al*, 2004). Very recently, indisulam was found to promote the production of reactive oxygen species before cellular apoptosis. Several de-hydrogenase enzymes have been identified so far as indisulam binding proteins, suggesting that the cellular redox control system connected with mitochondrial functions might be a potential primary target of indisulam (Oda *et al*, 2003).

A single agent phase II study revealed evidence of only weak activity and an acceptable toxicity profile in patients with non-small cell lung cancer (NSCLC), who had received prior treatment with platinum-based chemotherapy (Talbot *et al*, 2002).

Preclinical studies indicated that the combination of indisulam with platinum was synergistic in both cell line isobologram analysis and in NSCLC xenograft models, possibly by reducing intracellular glutathione (Ozawa *et al*, 2004). Sequencing carboplatin 1 day after the application of indisulam is based on experiments with human tumour xenografts in nude mice in which a significant downregulation of glutathione synthetase mRNA was detected by quantitative real-time polymerase chain reaction (RT-PCR) 24 h after administration of indisulam. As this enzyme is critical to maintain intracellular levels of glutathione that inactivates platinum species, this sequence guarantees non-compromised anti-tumour activity of carboplatin given on day 2 after indisulam (Owa T, Eisai Co Ltd, Japan; personal communication).

The currently presented dose escalation study was performed to identify a safe dose of indisulam in combination with carboplatin that can be tested in patients with NSCLC or other platinum-sensitive malignancies. For more than a decade, platinum-based chemotherapy represents the standard of care for NSCLC (Bunn *et al*, 1994). In a recently published prospectively randomised phase III study which compared four different platinum containing doublets, no significant difference with regard to response and survival was detected between three cisplatin- and one carboplatin-containing regimen (Schiller *et al*, 2002). The carboplatin-containing treatment arm was associated with even less toxicity overall, particularly with respect to febrile neutropenia and nausea. In a recent survey, a carboplatin-containing combination was found to be the most widely favoured option for first-line chemotherapy in all stages of NSCLC among US medical oncologists (Choy *et al*, 2000). This relates to ease of administration on an out-patient basis, manageable toxicity profiles compared with other platinum-containing regimens and promising phase II trial results (Langer *et al*, 1995; Muggia *et al*, 1995; Belani *et al*, 1996; Johnson *et al*, 1996).

The objectives of this study were (1) to determine the recommended dose of indisulam in combination with carboplatin in patients with solid tumours and (2) to evaluate the pharmacokinetics of the combination.

## PATIENTS AND METHODS

### Eligibility

Patients, ⩾18 years old, with a histologically/cytologically confirmed solid tumour refractory to standard therapy or for whom no established therapy exists were eligible. Their Karnofsky performance status must have been ⩾70%; they had to present with adequate haematological, renal and hepatic function as defined by absolute neutrophil count of ⩾1.5 × 10^9^ l^−1^; platelet count of ⩾100 × 10^9^ l^−1^; haemoglobin level of ⩾10g dl^−1^; serum bilirubin ⩽1.5 times the upper limit of normal, alanine aminotransferase (ALT) and aspartate aminotransferase (AST) ⩽2.5 times the upper limit of normal (⩽5 times the upper limit of normal in the presence of hepatic metastases); and serum creatinine ⩽1.5 times the upper limit of normal or a creatinine clearance ⩾60 ml min^−1^ (by Cockcroft-Gault formula).

Each of the following conditions represented an exclusion criterion: more than two previous lines of chemotherapy; incomplete recovery from surgery or radiation therapy; previous chemotherapy within 4 weeks of study entry; uncontrolled infections; significant cardiac impairment; and history of sensitivity to sulphonamides or platinum agents.

Written informed consent fulfilling the requirements of the ethics committee was required to be obtained from all patients before entry into the study.

### Treatment plan and study design

Indisulam dosing was based on body surface area and carboplatin doses were calculated with the Calvert formula to produce an area under the concentration–time curve (AUC) of 5 or 6 mg min ml^−1^ (Calvert *et al*, 1989; van Kesteren *et al*, 2002). The starting dose of the combination was indisulam 350 mg m^−2^ administered as a 1-h intravenous infusion on day 1 and carboplatin 6 mg min ml^−1^ as a 30-min infusion on day 2, repeated every 3 weeks. The dose escalation scheme is summarised in Table 1. The dose of indisulam was to be escalated in subsequent cohorts consisting of three patients to 500, 600 and 700 mg m^−2^ according to the toxicity observed at prior dose levels. If one of the first three patients experienced dose-limiting toxicity (DLT) during cycle 1, the number of patients treated at this dose level was expanded to six. Provided that none of the additional three patients experienced a DLT during cycle 1, dose escalation was continued. When a dose of indisulam was reached at which ⩾33% of the patients in a cohort of six experienced DLT during their first cycle of the combination, the dose of carboplatin was reduced from 6 to 5 mg min ml^−1^. If the maximum tolerated dose (MTD) of indisulam was well tolerated in combination with carboplatin 5 mg min ml^−1^, the indisulam dose was finally escalated to the next dose level.

### Patient evaluation and follow-up

Patients attended the clinic for a screening visit up to 14 days before treatment start to assess eligibility. Physical examination was performed as well as Karnofsky performance status was assessed at screening and vital signs as well as laboratory assessment (urinalysis, haematology, clinical chemistry) were carried out weekly till the final visit 30 days after the last intake of study drug. Patients were followed up for survival.

Tumour assessments according to RECIST criteria were carried out at screening and then every 6 weeks. ECGs were taken at screening and before and after study drug administration at day 1 only. Adverse events and concomitant medications were recorded on an ongoing basis from the date of consent until the final visit.

Dose limiting toxicities were assessed in the first cycle of treatment and were defined according to version 2.0 of the NCI common toxicity criteria (CTC): (i) ⩾grade 3 non-haematological toxicity directly related to study treatment (other than untreated nausea and vomiting), (ii) grade 4 thrombocytopenia, (iii) grade 4 neutropenia for ⩾7 days and (iv) ⩾grade 3 febrile neutropenia.

Dose reductions for unacceptable toxicity up to a maximum of two per patient were permitted. Once, the dose had been reduced it was not increased at a later date. A delay of up to 2 weeks was permitted in between cycles.

### Pharmacokinetic study

Full pharmacokinetic sampling of indisulam and carboplatin were performed during the first cycle of treatment. Blood samples (4 ml) for indisulam analysis were obtained at 15 time points for up to 8 days after the first administration: pre-infusion, 30 min after the start of infusion, at the end of the infusion, at 10 and 30 min and at 1, 2, 4, 6, 8, 24, 48, 72, 96 and 168 h after the end of the infusion. In addition, indisulam samples were taken before infusion and at the end of infusion during cycle 2. Immediately after collection, the samples were centrifuged at room temperature for 10 min at 1500 **g**. Plasma was collected and immediately stored at −20°C until analysis. The concentrations of indisulam in plasma were measured using high-performance liquid chromatography coupled to an electrospray ionization tandem mass spectrometer (LC/ESI-MS/MS) as described previously (Beumer *et al*, 2004).

Blood samples (5 ml) for carboplatin analysis were obtained at eight time points on day 1: before administration, at the end of infusion and at 1, 2, 4, 6, 8 and 23 h after the end of infusion. Immediately after collection, the samples were centrifuged at 4°C for 5 min at 1500 **g**. Immediately after centrifugation for plasma separation, aliquots of the collected plasma were transferred to Amicon ultrafiltration devices with an YMT-14 membrane (30 kDa MW cutoff; Millipore Corporation, Bedford, MA, USA). Ultrafiltrate was prepared by centrifuging this device at 1500 **g** for 15 min at room temperature. The plasma ultrafiltrates were stored frozen at −20°C until analysis of free platinum; the remaining plasma was stored frozen at −20°C until analysis of total platinum. Plasma concentrations were measured by graphite-furnace atomic-absorption spectrometry (van Warmerdam *et al*, 1995).

### Population pharmacokinetic analysis

The pharmacokinetic profile of indisulam monotherapy was described previously by a population pharmacokinetic model using nonlinear mixed effects modelling (Zandvliet *et al*, 2006). The model consisted of multiple compartments corresponding to plasma, erythrocytes, interstitial fluid and tissue. Indisulam was eliminated via two pathways: a linear and a saturable pathway. The pharmacokinetic parameters describing the model for indisulam monotherapy were applied to calculate model predicted indisulam concentrations, which were compared to the observed plasma concentration of indisulam. If the model adequately predicted the observed concentrations, it could be concluded that the pharmacokinetic profile of indisulam was not highly influenced by combination therapy with carboplatin. The population pharmacokinetic analyses were performed using NONMEM software (version V, level 1.1) (GloboMax LLC, Hanover, MD, USA) after logarithmic transformation of the data (Beal *et al*, 1988).

The pharmacokinetic results of ultrafiltrable carboplatin were also evaluated by compartmental analysis using NONMEM software. The data were fitted to a linear two-compartment model (Huitema *et al*, 2000; Shen *et al*, 2002; Ekhart *et al*, 2006). Pharmacokinetic parameters were compared to previously published reports (Huitema *et al*, 2000; Shen *et al*, 2002; Ekhart *et al*, 2006). Differences may indicate a pharmacokinetic interaction between carboplatin and indisulam. Furthermore, the observed carboplatin clearance of the studied patients, as determined by the population pharmacokinetic analysis, was compared to the predicted carboplatin clearance, as calculated using the Calvert formula (Eq. 1) (Calvert *et al*, 1989). Calvert *et al* (1989) demonstrated that the total plasma clearance of carboplatin was linearly related to the glomerular filtration rate (GFR) and that the non-renal clearance was 25 ml min^−1^. 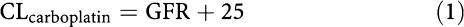


The estimated creatinine clearance (CL_cr_) was considered as a measure of GFR and was calculated from measured serum creatinine (CR, *μ*mol l^−1^), age (years), gender (0=male, 1=female) and weight (kg) using the Cockcroft and Gault's (1976) formula: 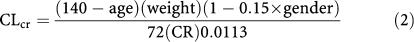


Serum creatinine levels were measured by enzymatic methods. If the observed carboplatin clearance corresponded well to the predictions from the Calvert formula, it could be concluded that the clearance of carboplatin was not affected by indisulam.

### Pharmacokinetic-pharmacodynamic analysis

Relationships between drug exposure and platelet counts were explored to find potential relationships between pharmacokinetics and haematological toxicity. The area under the plasma concentration *vs* time-curve (AUC) of indisulam and carboplatin ultrafiltrate were used as measures of drug exposure in this analysis. The relationship between the nadir platelet count and the exposure to indisulam (AUC_ind_) and carboplatin (AUC_carb_) at cycle 1 was described by a modified Hill equation: 
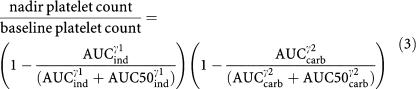


## RESULTS

### Dose escalation and safety assessment

Seventeen patients registered for the study; 16 patients received treatment with indisulam and carboplatin. Characteristics of eligible patients are listed in Table 2.

Patients were treated at four different dose levels (Table 3). Four patients were treated with 350 mg m^−2^ indisulam and 6 mg min ml^−1^ carboplatin. One patient died from progressive disease before completing 1 cycle of study treatment and was therefore replaced in the cohort. Three patients were treated at the second dose level. At the initial two dose levels, no DLTs were observed and the combination was well tolerated at cycle 1. However, all patients who received more than one treatment cycle required a delay in the start of subsequent cycles to recover from haematological toxicity. Four patients were treated at dose level 3. At cycle 1, two patients had dose-limiting grade 4 thrombocytopenia and one patient had grade 3 thrombocytopenia with haemorrhage (this was not defined as a DLT, but was considered as one by the investigator as it yielded consecutive partial liver resection owing to the intra-hepatic bleeding). A fourth patient in this cohort experienced CTC grade 4 neutropenia at cycle 3. The MTD of indisulam when administered in combination with carboplatin was consequently defined as 600 mg m^−2^. Consecutively, five patients were treated with 600 mg m^−2^ indisulam and 5 mg min ml^−1^ carboplatin. Two patients experienced DLTs at cycle 1 at this dose.

Overall, indisulam 600 mg m^−2^ in combination with carboplatin was too toxic. Nonetheless, a dose of 500 mg m^−2^ indisulam and carboplatin 6 mg min ml^−1^ was considered to be safe at cycle 1.

A total of 53 cycles of the combination were delivered, with a median of 2 cycles and a range of 1–7 cycles. Of 37 doses that were administered after the first treatment cycle, 30 (81%) were delayed by 1 or 2 weeks owing to unresolved thrombocytopenia and/or neutropenia.

Sixteen patients were evaluated for toxicity. Table 4a represents a summary of treatment-related adverse events. Nearly all severe adverse events were haematological toxicities (thrombocytopenia, neutropenia and anaemia) occurring by day 15 of each cycle, and resolving after at least 7 days. Table 4b shows the number of patients with haematological toxicity CTC grade 3 or 4 at cycle 1 and post cycle 1. Thrombocytopenia was the major DLT at cycle 1. Furthermore, all patients who received more than one treatment cycle experienced thrombocytopenia as a result of the combination treatment with indisulam and carboplatin. Non-haematological side effects were minor and can be neglected.

Severe adverse events related to study treatment reported during the study were: grade 4 thrombocytopenia (2), grade 3 thrombocytopenia with bleeding (1), grade 4 neutropenia (1), grade 4 anaemia (1) and grade 2 supra-ventricular tachycardia (1).

Three patients died on study (within 30 days of last study treatment) due to progressive disease. Six patients withdrew due to adverse events, four due to progressive disease according to RECIST criteria, three due to clinical progressive disease, one due to abnormal laboratory values (neutropenia grade 3) and one withdrew consent.

### Response

Ten patients completed at least two cycles of study treatment and were evaluable for response. Eight patients showed stable disease and two patients had progressive disease.

### Population pharmacokinetic analysis

**Figure 1:**
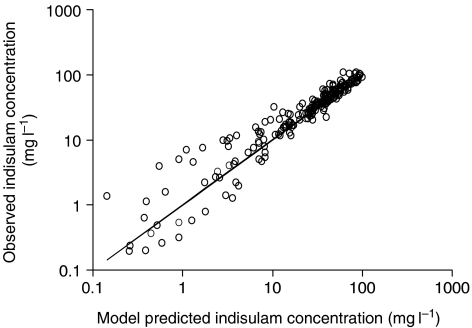
Observed *vs* model predicted plasma concentrations of indisulam. The scatter plot is symmetrically positioned around the line of identity, which indicates that the currently observed profiles are well described by the previously developed population pharmacokinetic model.

Sixteen patients were evaluable for pharmacokinetics. Time profiles of indisulam plasma concentrations were adequately described by the population pharmacokinetic model. Observed indisulam plasma concentrations corresponded well to the model-based predictions (Figure 1). The median exposures to indisulam expressed as the AUC were 47, 94 and 116 mg min ml^−1^ for dose levels 350, 500 and 600 mg m^−2^ and AUCs ranged from 25 to 198 mg min ml^−1^.

**Figure 2:**
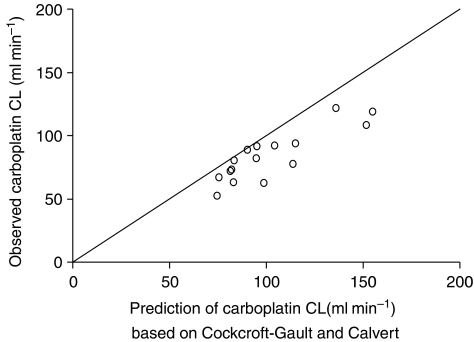
Observed carboplatin clearance (CL) *vs* predicted carboplatin clearance based on Cockcroft and Gault (1976) and Calvert *et al* (1989).

The two-compartmental model produced a good fit to the ultrafiltrable carboplatin concentrations. Estimated pharmacokinetic parameters are listed in Table 5. Carboplatin clearance was lower than in previous studies. Conversely, the intercompartmental clearance was relatively high. Other parameters corresponded well to published values. Figure 2 shows that the carboplatin clearance was overpredicted by the Calvert formula for all patients. This resulted in a carboplatin exposure that was higher than the target exposure. The median exposure was 6.1 mg min ml^−1^ for target 5 mg min ml^−1^ and 6.7 mg min ml^−1^ for target 6 mg min ml^−1^. Carboplatin AUC ranged from 5 to 10 mg min ml^−1^.

### Pharmacokinetic-pharmacodynamic analysis

**Figure 3:**
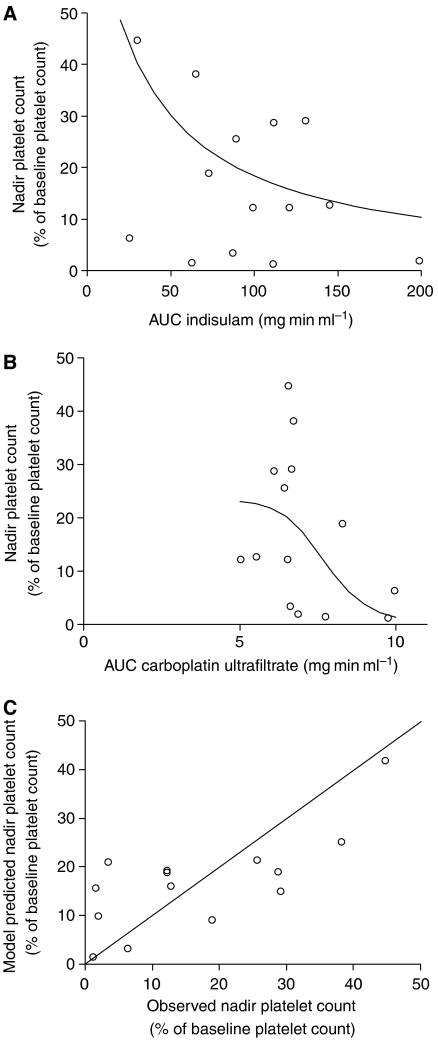
The relative nadir platelet count *vs* exposure to indisulam (**A**) and exposure to carboplatin (**B**). The solid lines represent the model predicted nadir counts after median exposure to carboplatin (6.7 mg min ml^−1^) and varying exposure to indisulam (**A**) or after median exposure to indisulam (94 mg min ml^−1^) and varying exposure to carboplatin (**B**). A plot of model predicted values *vs* the corresponding observed values (**C**) is symmetrically distributed around the line of unity, which demonstrates that the modified Hill equation resulted in unbiased predictions of the nadir platelet count. AUC, area under the plasma concentration *vs* time curve.

The severity of thrombocytopenia was expressed as the nadir platelet count and was related to the exposure to indisulam and carboplatin. Myelosuppression was assessed at the first treatment cycle. Fourteen patients were evaluable for this PK-PD analysis. Their nadir platelet count ranged from 4 × 10^9^ to 154 × 10^9^ l^−1^ corresponding to a range of 1.2–45% of the baseline platelet count. The relative nadir platelet count, expressed as a percentage of the baseline platelet count, decreased with both indisulam AUC (*P*=0.003) and carboplatin AUC (*P*=0.004). The AUC of indisulam resulting in a 50% decrease in platelet count (AUC50_ind_ in Eq. 3) was estimated at 28.5 mg min ml^−1^ (relative standard error (RSE) 21%). The thrombocytopenic effect of carboplatin was half maximal at an AUC of 7.74 mg min ml^−1^ (RSE 13%) (AUC50_carb_ in Eq. 3). The Hill factor *γ*1 was not significantly different from 1 (*P*=0.57). The myelosuppressive effect of carboplatin was best described by a sigmoidal E_max_ model and *γ*^2^ was estimated at 10.9 (RSE 70%). The relationship between the relative nadir platelet count and the exposure to indisulam and carboplatin is depicted in Figure 3A and B. The model predicted nadir platelet counts were unbiased and corresponded reasonably well to the observed values (Figure 3C). The modified Hill equation adequately described the relative nadir platelet count after treatment with indisulam in combination with carboplatin.

## DISCUSSION

Both, the weak anti-tumour activity of indisulam as single agent at a dosage of 700 mg m^−2^ as intravenous infusion over 1 h every 3 weeks, demonstrated by tumour regressions of lesser degree than required for an objective response in about third of the patients treated and the low rate of toxicity with less than a quarter of all patients experiencing grade 3 or 4 haematological toxicity, which represented the main toxicity, did not only justify but merely prompted us to combine indisulam with a substance with known anti-tumour activity against NSCLC (Talbot *et al*, 2002). As a consequence, carboplatin was selected (Bunn *et al*, 1994; Langer *et al*, 1995; Muggia *et al*, 1995; Belani *et al*, 1996; Johnson *et al*, 1996; Choy *et al*, 2000; Schiller *et al*, 2002).

Haematological toxicity was dose limiting for indisulam in combination with carboplatin. Minimal non-haematological toxicity was observed after treatment with the combination.

A dose of 500 mg m^−2^ indisulam and 6 mg min ml^−1^ carboplatin was well tolerated at cycle 1. However, dose delays were common due to prolonged marrow recovery from the previous treatment. Therefore, a 3-weekly treatment cycle was not sustainable. Thus, for a future phase II study with indisulam and carboplatin in chemotherapy naïve patients with NSCLC, a 4-weekly schedule was proposed. In this phase II study, patients will be treated with the safe dose of 500 mg m^−2^ indisulam with 6 mg min ml^−1^ carboplatin at cycle 1. Patients with minor thrombocytopenia (less than grade 3) at cycle 1 may receive an escalated dose of indisulam of 600 mg m^−2^ at cycle 2, because previous studies have demonstrated a high correlation between the extent of prior chemotherapy and the incidence of haematological toxicity.

Myelosuppression was previously identified as the DLT for single agent therapy with both indisulam and carboplatin (Calvert *et al*, 1982; Raymond *et al*, 2002). Thrombocytopenia was identified as the major toxicity of the combination. The nadir platelet count decreased with increasing exposure to both indisulam and carboplatin. This phenomenon can be interpreted as the result of the addition of a similar behaviour of the two substances by increasing their dosages separately, but not primarily as the result of its pharmacodynamic interaction. Single agent high dose carboplatin, that is, 520–1000 mg m^−2^, covering the exposure of an AUC ranging from 5 to 10 mg min ml^−1^, which was reached in our study, yielded severe and cumulative bone marrow suppression in ovarian cancer patients (Wiltshaw *et al*, 1987). Both, leucopenia and thrombocytopenia reached their nadirs at day 15; leucopenia <1000 leucocytes *μ*l^−1^ lasted 4 days (range 1–11 days), thrombocytopenia <100 000 platelets *μ*l^−1^ lasted 12 days (range 4–40 days). Recommendations for target AUC for obtaining an acceptable degree of myelosuppression resulted in an AUC of 5–7 mg min ml^−1^ for carboplatin single agent therapy and in an AUC of 4–5 mg min ml^−1^, when the drug was to be used in combination with other myelosuppressive agents (Calvert *et al*, 1989). In a phase II study in which carboplatin was tested at an AUC of 7 mg min ml^−1^ – corresponding well with the median exposure of 6.7 mg min ml^−1^ for target AUC 6 mg min ml^−1^ in our study – every 4 weeks in advanced breast cancer patients, leucopenia at the time of scheduled re-treatment was found to be of grade 3 in 6% of the patients, of grade 2 in 15% and of grade 1 in 22%; the respective value for thrombocytopenia was grade 3 in 5% of the patients (O'Brien *et al*, 1993). Martin *et al* (1992), who tested a dosage of only 400 mg m^−2^, every 4 weeks, in metastatic breast cancer patients, observed only infrequent and mild leucopenia and thrombocytopenia on day 28. Overall, leucopenia of only WHO grade 1–2 was found in 47% of the patients or 18% of the courses and thrombocytopenia in 12% of the patients or 3% of the courses, respectively. A scarce trial in which carboplatin was tested at a 3-weekly period, as foreseen in our study, but at a dosage of only 400 mg m^−2^ in metastatic breast cancer patients, yielded leucopenia grade 3–4 in two out of 20 (10%) patients and thrombocytopenia grade 3 in one out of 20 (5%) patients as worst haematological toxicities. But, due to permanent myelosuppression, five cycles of carboplatin could be administered to only three patients (Kolarić and Vukas, 1991). Overall, the prolonged cytopenia concerning both platelets and neutrophils, which was observed in our study, can be explained on the basis of exposure to single agent carboplatin.

Conversely, these toxicities are at least not caused by indisulam single agent therapy. Thrombocytopenia usually occurred later than leucopenia between days 9 and 14 and was of short duration (median 3 days; range 1–6 days). Leucopenia typically occurred at day 8 (range days 7–12) of the first cycle, and was associated with neutropenia at day 10 (range days 6–11), lasted for a median period of 8 days (range 7–9 days) and was fully reversible. Of the 21 cycles of indisulam given at the 700 mg m^−2^ dose level as 1 h infusion, every 3 weeks, to heavily pretreated patients, grade 3 or 4 neutropenia, thrombocytopenia or anaemia was reported in two cycles (9.5%), three cycles (14%) and one cycle (4.7%), respectively (Raymond *et al*, 2002).

The exposure to carboplatin was higher than anticipated in all patients owing to overestimation of the carboplatin clearance by application of the Calvert formula (with GFR estimated by CL_cr_ derived from Cockcroft-Gault). Consequently, higher doses than required to reach the predefined AUC values were administered. This has contributed to the high level of haematological toxicity that was observed in this study. In addition to the relatively high exposure to carboplatin, a pharmacodynamic interaction with indisulam may have caused excessive haematological toxicity in this study. Indisulam reduces the transcription of glutathione transcriptase and glutathione reductase resulting in a reduction of intracellular glutathione levels. This may not only enhance anti-tumour activity of carboplatin, but it may also increase haematological toxicity. This hypothesis is supported by the effect of L-carnitine, which protects against carboplatin-induced myelosuppression by increasing the intracellular glutathione content during treatment with carboplatin (Abd-Allah *et al*, 2005).

Obviously, it cannot be excluded, but is merely to be expected that the synergism determined for the combination of the two drugs in preclinical experiments is the reason for the observed prolonged cytopenia of both platelets and neutrophils as observed in our study.

The pharmacokinetic characteristics of indisulam were not significantly affected by carboplatin, which was demonstrated by the lack of bias in Figure 1. Carboplatin clearance was lower than previously reported values and consistently lower than predicted by the Calvert formula (Figure 2). It is unlikely that indisulam affects renal elimination of carboplatin, because only seven out of 391 patients that had previously been treated with indisulam had renal dysfunction. More probably, the low carboplatin clearance was related to overestimation of the GFR by two mechanisms. Serum creatinine is often assumed to be eliminated by passive renal elimination only. However, active tubular secretion accounts for about 20% of creatinine clearance. The tubular secretion may be counterbalanced by overestimation of the serum creatinine level as a result of the interference of non-creatinine chromogens with the alkaline picrate method of Jaffe (Diskin, 2006). Conversely, in our study, serum creatinine levels were measured by more accurate enzymatic methods. The consequent lack of a compensation for the tubular secretion of creatinine may have caused an overestimation of the GFR and consequently of the carboplatin clearance.

In conclusion, this study showed that the combination of indisulam with carboplatin was safe and a MTD for the phase II study was identified. The three-weekly treatment schedule was not feasible owing to thrombocytopenia and was therefore extended to a four-weekly regimen.

## Figures and Tables

**Table 1:** Summary of planned dose escalation scheme

**Dose level**	**Indisulam (mg m^−2^) day 1**	**Carboplatin (mg min ml^−1^) day 2**
One	350	6
Two	500	6
Three	600	6
Four	700	6
*X*	Maximum tolerated dose	5
*X*+1	Maximum tolerated dose + one	5

**Table 2:** Patient characteristics

*Gender*	
Male	5 (31%)
Female	11 (69%)
	
*Age (years)*	
Median	63
Range	19–81
	
*Height (cm)*	
Median	164
Range	153–185
	
*Race*	
Caucasian	16 (100%)
	
*Time since diagnosis (months)*	
Median	30
Range	2–335
	
*Tumour type*	
Sarcoma	3
Renal cell	2
Lung	2
Colorectal	2
Ovarian	2
Pancreas	2
Adenocarcinoma	1
Melanoma	1
Gastric	1
	
*Previous therapy*	
Surgery	16 (100%)
Radiotherapy	5 (31%)
Chemotherapy, 1 course	5 (31%)
Chemotherapy, 2 courses	4 (25%)
Chemotherapy, 3 courses	1 (6%)
Chemotherapy, 4 courses	2 (13%)
Experimental therapy	4 (25%)

**Table 3:** Number of patients, DLTs, dose reductions per dose level

**Indisulam (mg m^−2^)**	**Carboplatin (mg min ml^−1^)**	**Number of patients treated**	**DLTs**	**Patients with dose reductions at cycle 2**
350	6	4^a^	0	0
500	6	3	0	0
600	6	4	3 (75%)	2
600	5	5^b^	2 (50%)^c^	0

DLTs, dose limiting toxicities.

a One patient was not assessable for DLT, because he died due to progressive disease during cycle 1.

b One patient was not assessable for DLT, because she died due to progressive disease during cycle 1

c Two DLTs out of four assessable patients.

**Table 4a:** Treatment-related adverse events observed during the study by worst grade according to the NCI-common toxicity criteria (CTC)

**Dose level^a^**	**350/6**		**500/6**		**600/6**		**600/5**		**All**	
**Number of evaluable patients**	**4**		**3**		**4**		**5**		**16**	
**CTC grade**	**1–2**	**3–4**	**1–2**	**3–4**	**1–2**	**3–4**	**1–2**	**3–4**	**1–2**	**3–4**
*Haematological adverse events*										
Thrombocytopenia	0	3	0	2	1	2	0	4	1 (6%)	11 (69%)
Neutropenia	1	2	1	1	0	3	0	4	2 (12.5%)	10 (62.5%)
Anaemia	2	1	1	1	0	3	1	2	4 (25%)	7 (44%)
										
*Non-haematological adverse events*										
Fatigue	3	0	3	0	3	0	4	0	13 (81%)	0 (0%)
Nausea	2	0	2	0	2	0	2	0	8 (50%)	0 (0%)
Vomiting	3	0	0	0	2	0	1	0	6 (37.5%)	0 (0%)
Constipation	2	0	1	0	3	0	0	0	6 (37.5%)	0 (0%)
Dysgeusia	0	0	1	0	1	0	2	0	4 (25%)	0 (0%)
Anorexia	1	0	1	0	1	0	1	0	4 (25%)	0 (0%)
Abdominal pain	2	0	0	0	2	0	0	0	4 (25%)	0 (0%)
Dry mouth	1	0	0	0	1	0	1	0	3 (19%)	0 (0%)
Flatulence	1	0	1	0	1	0	0	0	3 (19%)	0 (0%)
Diarrhoea	1	0	0	0	1	0	0	0	2 (12.5%)	0 (0%)
Headache	1	0	0	0	1	0	0	0	2 (12.5%)	0 (0%)
Dyspepsia	1	0	1	0	0	0	0	0	2 (12.5%)	0 (0%)
Peripheral oedema	0	0	1	0	0	0	1	0	2 (12.5%)	0 (0%)
Dry skin	1	0	1	0	0	0	0	0	2 (12.5%)	0 (0%)

a Indisulam (mg m^−2^)/carboplatin (mg min ml^−1^).

**Table 4b:** Number of patients with any treatment-related haematological toxicity grade 3 or 4 according to the NCI-common toxicity criteria (CTC) at cycle 1 and post cycle 1

**Dose level^a^**	**350/6**		**500/6**		**600/6**		**600/5**		**All**	
**Number of evaluable patients**	**4**		**3**		**4**		**5**		**16**	
**CTC grade**	**3**	**4**	**3**	**4**	**3**	**4**	**3**	**4**	**3**	**4**
*Cycle 1*										
Platelets	0	0	1	0	1	2	3	1	5 (31%)	3 (19%)
Neutrophils	0	0	0	0	1	1	0	1	1 (6%)	2 (13%)
Leucocytes	0	0	0	0	1	0	1	1	2 (13%)	1 (6%)
Lymphocytes	0	0	0	0	0	0	1	0	1 (6%)	0 (0%)
Haemoglobin	0	0	0	0	0	1	2	0	2 (13%)	1 (6%)
										
*Post cycle 1*										
Platelets	3	0	2	1	1	2	4	1	10 (63%)	4 (25%)
Neutrophils	2	0	2	1	3	2	2	4	9 (56%)	7 (44%)
Leucocytes	2	0	1	0	1	0	4	2	8 (50%)	2 (13%)
Lymphocytes	1	0	0	0	0	0	3	0	4 (25%)	0 (0%)
Haemoglobin	2	0	1	0	2	0	2	0	7 (44%)	0 (0%)

a Indisulam (mg m^−2^)/carboplatin (mg min ml^−1^).

**Table 5:** Population pharmacokinetic parameter estimates of carboplatin ultrafiltrate

**Pharmacokinetic parameter**	**Previous reports^a^**	**Current estimate**	**(%SE)**	**IIV%**	**(%SE)**
Clearance CL (l h^−1^)	6.06–8.33	4.87	(6)	12.4	(29)
Central volume of distribution V1 (l)	14.4–16.3	15.5	(20)	66.2	(147)
Intercompartmental clearance Q (l h^−1^)	0.792–1.70	3.46	(18)	28.3	(39)
Peripheral volume of distribution V2 (l)	7.07–10.4	9.94	(11)	19.8	(41)
Correlation coefficientρ CL∼V1		0.67			
Proportional residual error (%)		9.50	(11)		

SE, standard error; IIV, interindividual variability.

a Huitema *et al*, (2000); Shen *et al*, (2002); Ekhart *et al*, (2006).
